# Structural and functional correlates for language efficiency in auditory word processing

**DOI:** 10.1371/journal.pone.0184232

**Published:** 2017-09-11

**Authors:** JeYoung Jung, Sunmi Kim, Hyesuk Cho, Kichun Nam

**Affiliations:** 1 Neuroscience and Aphasia Research Unit, Division of Neuroscience & Experimental Psychology, School of Biological Sciences, University of Manchester, Manchester, United Kingdom; 2 Wisdom Science Centre, Korea University, Seoul, South Korea; 3 Department of Psychology, Korea University, Seoul, South Korea; Hangzhou Normal University, CHINA

## Abstract

This study aims to provide convergent understanding of the neural basis of auditory word processing efficiency using a multimodal imaging. We investigated the structural and functional correlates of word processing efficiency in healthy individuals. We acquired two structural imaging (T1-weighted imaging and diffusion tensor imaging) and functional magnetic resonance imaging (fMRI) during auditory word processing (phonological and semantic tasks). Our results showed that better phonological performance was predicted by the greater thalamus activity. In contrary, better semantic performance was associated with the less activation in the left posterior middle temporal gyrus (pMTG), supporting the neural efficiency hypothesis that better task performance requires less brain activation. Furthermore, our network analysis revealed the semantic network including the left anterior temporal lobe (ATL), dorsolateral prefrontal cortex (DLPFC) and pMTG was correlated with the semantic efficiency. Especially, this network acted as a neural efficient manner during auditory word processing. Structurally, DLPFC and cingulum contributed to the word processing efficiency. Also, the parietal cortex showed a significate association with the word processing efficiency. Our results demonstrated that two features of word processing efficiency, phonology and semantics, can be supported in different brain regions and, importantly, the way serving it in each region was different according to the feature of word processing. Our findings suggest that word processing efficiency can be achieved by in collaboration of multiple brain regions involved in language and general cognitive function structurally and functionally.

## Introduction

Word processing is a key component of language comprehension. Accumulating evidence from neurological and functional brain imaging studies has suggested that word processing is involved in the left lateralized large-scale of brain network including frontal, temporal, and parietal regions [1–4]. For successful comprehension in communication, it requires to retrieve and process word timely, which is described as word processing efficiency. Word processing efficiency varies across individuals and may be attributed to increase or decrease activity of brain regions in the distributed language network. Although it is important in our daily communication, the underlying neural mechanisms of word processing efficiency still remain unclear.

Only a few studies have addressed the relationship between brain activity and word processing efficiency on language functions using fMRI. Booth and colleagues [5] showed modality-specific regional activity related to language performance. Individuals with high performance on a visual spelling task had increased activation in the fusiform gyrus, whereas individuals with high performance on an auditory rhyming task showed the increased activation in the bilateral superior temporal gyrus, the left supramarginal gyrus, and angular gyrus. A study with presurgical epilepsy patients demonstrated that their performance on a semantic decision task was positively correlated with the activity of the left temporo-parietal regions [6]. Wei and colleagues [7] found that the blood oxygen level-dependent (BOLD) signal of the left posterior middle temporal gyrus (pMTG) predicted participants’ semantic processing efficiency at rest. Another study by Chee and colleagues [8] investigated brain activation in Chinese-English bilinguals by linking with their performance. They reported that better performing participants exhibited the decreased activation in the left IFG and left parietal lobe, supporting the neural efficiency hypothesis [9] that better tuned neuronal networks predict enhanced performance and thus less neuronal activity is required to perform the task. These studies indicate that brain regions related to language function including frontal, temporal and parietal areas can be involved in word processing efficiency. However, fMRI findings have showed various and somewhat inconsistent results of the locus of brain and its regional activity.

Structural magnetic resonance imaging studies of language using voxel-based morphometry (VBM), [10] and diffusion tensor imaging (DTI), [11] have localized brain regions on language functions by comparing regional involvements across two groups of participants (e.g., trained or untrained) or correlating of brain structure with language ability. Several studies have showed that lexical efficiency is associated with the gray matter density of the left frontal, parietal, and temporal regions for both trained and untrained individuals [12–15]. A study [13] demonstrated a positive correlation between word knowledge and gray matter density of bilateral supramarginal gyrus in adolescents. Richardson and colleagues [15] showed a positive correlation between gray matter of the left temporal regions and word knowledge in adult. In DTI literature, it has been reported repetitively that the higher value of fractional anisotropy (FA) in the left arcuate fasciculus predicted better performance in reading [16–19]. Structural studies demonstrated that the structural features (gray and white matter density) of multiple language regions were associated with word processing efficiency. Although MRI studies suggest that brain regions related with language are involved in individual performance in language functions, there has been no consensus about which and how brain structure plays a critical role in word efficiency. These discordances may arise from differences in tasks, neural measurements or subject groups (e.g., trained vs. untrained or young vs. old). Thus, here, we aimed to examine both structural and functional neural aspects of word processing efficiency in the healthy participants. Especially, we employed two different tasks to tackle key aspects of word processing: phonology and semantics.

In the current study, we investigated both functional and structural correlates of word processing efficiency for phonological and semantic processing. We examined how brain activity (task-induced regional activity and functional connectivity) and brain structural measures (gray matter density and white matter FA) predicts individual variations in auditory word processing capacity during the phonological and semantic processing: (1) we performed correlation analyses between participants’ task performance and their regional BOLD signal changes; (2) we defined the core region as seed and calculated functional connectivity between the seed and other voxels to establish the brain network associated with each word processing efficiency; (3) we performed correlation analyses between regions of interest (ROIs, brain regions and pathways related to language function) and each word processing efficiency. We hypothesized that the word processing efficiency would be involved in the subsets of language and that two aspects of language efficiency (phonology and semantics) would be served in different sets of these regions.

## Materials and methods

### Participants

Prior to the experiment, we performed a power analysis to decide the number of participants in order to obtain 80% of power in a correlation analysis (p < 0.05, two-tailed, expected r = 0.6) and the result showed fourteen participants were enough to achieve the intended statistical power [20]. Based on the prior power analysis, we recruited sixteen participants for this study. Data were collected from sixteen, right-handed native Korean speaking participants (7 males and 9 females, mean age: 23.4 ± 2.5years). Participants had normal hearing and no neurological history. Written consent forms were obtained from all participants prior to the experiment. This study was approved by the Ethical Committee of Korea University.

### Stimulus

194 words were selected based on phonological and semantic features from a Korean pronunciation dictionary [21]. We conducted a familiarity questionnaire with 7 points Likert scale for the selected words. Thirty Korean native speakers answered the questionnaire and evaluated the emotional valence with three choices (negative, neutral and positive). A total of 60 nouns scored 7 point (very familiar) at the questionnaire were selected and recorded by two voice actors (a male and a female) (S1 Table). The average duration of words was 1.08s ± 0.23s. All nouns consisted of two syllables with high frequency. The half of nouns in the list has a long vowel at the first syllable. Korean has a vowel-length contrast in words [21–23]. For example, 눈nwun means either ‘snow’ or ‘eye’. When its vowel is pronounced short, [nun], it means ‘eye’, whereas when pronounced long, [nu:n], it means ‘snow’. Such long vowels can typically occur in the first syllable of a word. In this study, we selected two syllable nouns having a long vowel in their first syllable without changing the meaning of words. The word list for the phonological judgement task consisted of 30 nouns (neutral meaning words) that the half of nouns had a long vowel at the first syllable and the others not. Words used in the semantic judgement task were 30 nouns, 15 positive and 15 negative meaning nouns.

### Magnetic resonance imaging

Scanning was performed at a 3 T Siemens scanner (Erlangen, Germany) in the Korea University Magnetic Resonance Imaging Centre. T1-weighted images were acquired using MP-RAGE sequence (TR/TE = 1900/2.52ms, flip angle = 9°, voxel size = 1 x 1 x 1 mm^3^) covering the whole head. Diffusion tensor images were acquired using a single-shot spin-echo, echo planar imaging (EPI) sequence in 60 noncollinear directions covering the whole head (TR/TE = 6300/84ms, voxel size = 1.8 x 1.8 x 3mm^3^, b values = 600s/mm^2^, SENSE acceleration factor = 2, number of slices = 50). Functional images were acquired using single-shot echo planer EPI sequences (TR/TE = 2000/30ms, flip angle = 90°, voxel size = 3 x 3 x 4 mm^3^, number of slices = 36).

**Fig 1:**
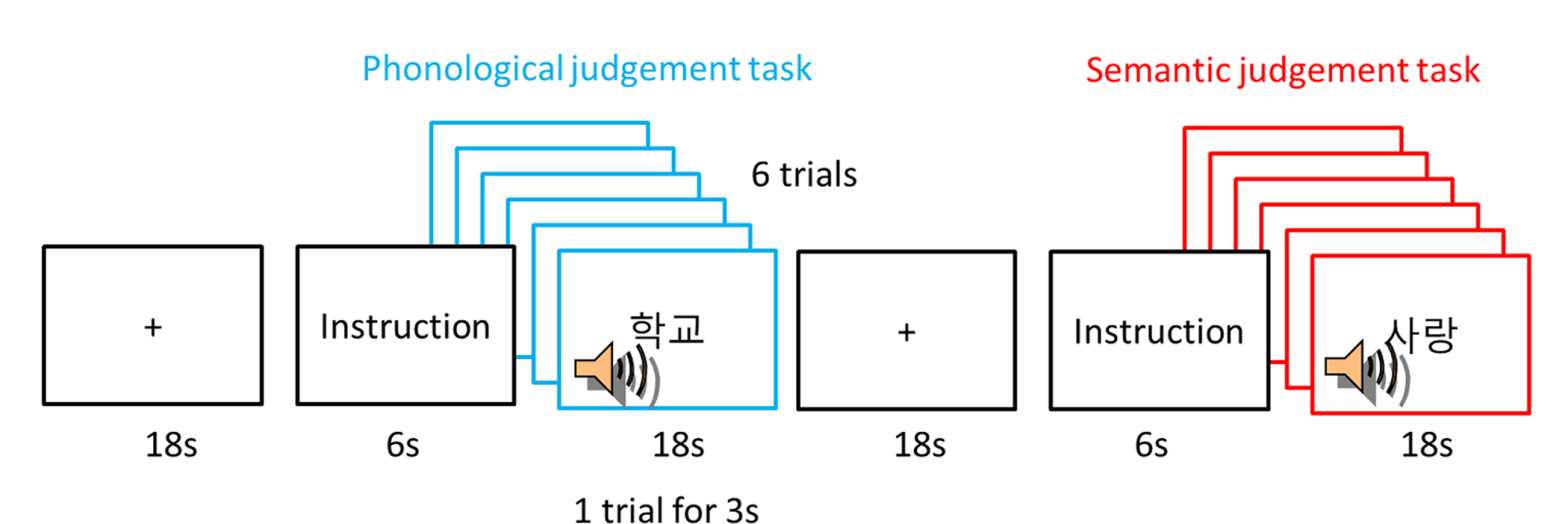
Experimental design.

In a block-designed fMRI session, two tasks were presented as a task-specific block. The session consisted of 5 blocks of the phonological judgement task and 5 blocks of the semantic judgement task interspersed with 10 fixation blocks (Fig 1). Fixations were presented for 18s before the 6s of an instruction. During the fixation, participants were asked to fixate a cross at the centre of the screen. The instruction indicated which task participants should perform in a following block. In a task block, 6 stimuli were heard through a headset for the duration of 18s and there was nothing presented on the screen. Participants should press a button with their right index finger according to the preceded instruction. In the phonological judgement task, participants were asked to press the first button if the first syllable had a long vowel and the second button if it did not. In the semantic judgement task, participants assessed the emotional valence of words (positive vs. negative). If the meaning was positive, they had to press the first button and the second button if the meaning was negative. The order of blocks was counterbalanced. The experiment was presented using E-Prime software.

### Behavioural analysis

To assess participants’ performance accounting for speed-accuracy trade-off effects, we calculated an inverse efficiency score in the analysis of behavioural data. The efficiency measure was acquired by dividing the average response time of correct trials by accuracy [24]. According to the tasks, we had phonological efficiency (PE) and semantic efficiency (SE). These inverse efficiency values were used as the index for participants’ word processing performance, that is lower value indicated better performance. S1 Fig shows the individual performance of each task.

### fMRI data analysis

The data were analysed using SPM8 (Wellcome Department of Imaging Neuroscience, www.fil.ion.ucl.ac.uk/spm) to investigate brain activation during auditory word processing. First, head motion correction was performed, and a mean functional image was obtained for each participant. Each participant’s structural image was coregistered to the mean functional image and segmented. Then the functional images were normalized to the Montreal Neurological Institute (MNI) template using parameters acquired in segmentation. Finally, all images were smoothed with an 8mm full-width half-maximal (FWHM) Gaussian kernel to decrease spatial noise. Statistical analyses were performed using a general linear model (GLM). Four contrasts were computed for each participant: phonological processing > fixation (PP > Fix); semantic processing > fixation (SP > Fix); phonological processing > semantic processing (PP > SP); semantic processing > phonological processing (SP >PP). In random-effects analyses, the contrasts images were entered into one-sample t-test to identify regions activated by task conditions. Multiple regression analyses were conducted for the contrast of PP > Fix and SP > Fix with PE and SE as regressors of interest each in order to find which region shows changes in the level of activity in relation to word processing efficiency (PE or SE). Gender was included as a nuisance variable. Statistical threshold was set at p < 0.005 at the voxel level and p < 0.05 at the cluster level with at least 30 contiguous voxels after false discovery rate (FDR) correction for multiple comparisons. SPM Anatomy Toolbox [25] was used to label the activated brain regions.

In order to confirm our results, we used the published data [26], which employed an auditory control task. In the task, twenty participants were asked to indicate button press whether the item was high or low sound during fMRI. The auditory stimuli were either high or low sounding noise burst (i.e., pink vs. brown noise). Then, we performed correlation analyses with each task performance (PE and SE) after comparing our task conditions with the control task (two-sample t-tests: phonology > control and semantics > control). The results demonstrated the same results as our original analyses (S2 Fig).

### Connectivity analysis

To demonstrated core brain areas related to PE and SE, we further explored the data based on the results of GLM analyses. We used the Functional Connectivity (CONN) Toolbox (http://web.mit.edu/swg/software.htm) to perform functional connectivity analysis. This toolbox achieves seed-based analysis by computing temporal correlation between BOLD signals from a voxel to all other voxels in the brain [27]. The seed area was defined by the result from the GLM analyses (thalamus and pMTG). Pre-processed images were registered in the toolbox with the seed. Connectivity analysis between BOLD signals from the seed and from the other brain voxels provided seed-to-voxel connectivity estimations for experimental conditions (Fix, PP, and SP). At the individual level analysis, head movements were entered as regressors. Before averaging individual data, all voxels were filtered using a band pass filter (0.01 <*f*<Inf) to decrease the effect of low-frequency drift. CompCor strategy implemented in the toolbox removed several sources of noise from white matter, cerebral fluid, and the others. For random-effects analyses, PE and SE were employed as regressors. Statistical threshold was defined at p < 0.05 FDR corrected with at least 30 contiguous voxels.

### Voxel-based morphometry (VBM)

VBM was performed to explore the relationship between the gray matter and participants’ word processing efficiency using VBM8 toolbox (http://dbm.neuro.uni-jena.de/vbm8/) implemented on SPM8. All T1-weight images were spatially normalized to SPM’s T1 template and segmented into gray matter, white matter, and cerebrospinal fluid. Diffeomorphic Anatomical Registration Through Exponentiated Lie Algebra (DARTEL) protocol was used for normalization [28]. During segmentation, a DARTEL template was created and each participant’s image was registered onto the template. Gray matter images were modulated and smoothed with an 8mm FWHM Gaussian kernel. Multiple regression models were established for PE and SE with covariates including total intracranial volume (TIV), age, and gender. We reported the results at p < 0.005 uncorrected. For structural imaging, it is difficult to find significant correlation with less than twenty participants [29]. As we had only sixteen participants in this study, we applied the less stringent threshold (p < 0.005 uncorrected) in order to identify brain structure correlated with word processing efficiency.

### Tract-based spatial statistics (TBSS)

DTI images were pre-processed to generate fractional antisotropy (FA) maps using FSL’s Diffusion Toolbox [30]. Each participant’s brain was extracted and corrected for eddy current distortion. The diffusion tensor models were fitted to the data and FA images were created. The FA images were aligned and transformed into MNI space using nonlinear registration tool FNIRT. Then a mean FA skeleton was created from the mean FA image, which represents the centre of all tracts. Each participant’s FA data was projected onto the skeleton. For statistical analyse, TBSS was used for the GLM with randomized permutation methods [31]. Correlation analyses were performed with PE and SE. We reported the results at p < 0.005 uncorrected.

### Region-of-interest (ROI) analysis

Language related brain areas were defined as ROIs to examine their involvements of word processing efficiency including frontal and parietal cortex [32]. These included pars opercularis (BA 44), pars triangularis (BA 45), pars orbitalis (BA 47), dorsolateral prefrontal cortex (BA 9 and BA 46), angular gyrus (BA 39), and supramarginal gyrus (BA 40). We used ROI templates from Wake Forest University Pickatlas toolbox [33] utilizing Brodmann’s areas. Four white matter tracts connecting temporal lobe to fronto-parietal language network from JHU DTI-based white-matter atlases [34] were selected: uncinate fasciculus, external capsule, superior longitudinal fasciculus, and sagittal stratum. For all ROIs, we extracted BOLD signals, gray matter density, and FA value from fMRI, VBM, and DTI data and correlated with efficiency values. Pearson’s correlation analyses were conducted with the significant level at p < 0.05 (two-tailed). Correlation coefficients for PE and SE were compared to determine if they were significantly different from each other [35].

## Results

### Behavioural results

Participants’ performance for the behavioural tasks is displayed in Table 1. Participants were faster in the phonological task than the semantic task (t(15) = -4.05, p < 0.001). Accuracy in the phonological processing was higher than in the semantic processing (t(15) = 2.25, p < 0.05). As a result, the inverse efficiency of the phonological task was smaller than that of semantic task (t(15) = -4.48, p < 0.001). The correlation analysis showed that each inverse efficiency was highly correlated with each other (r = 0.66, p < 0.01). Participants showing high performance in the phonological task also performed the semantic task efficiently (S1 Fig).

**Table 1:** Participants’ behavioural performance.

	Reaction time (ms)		Accuray (%)		Inverse efficiency	
	Mean	SD	Mean	SD	Mean	SD
Phonological task	451.0	170.8	94.1	3.3	481.8	190.4
Semantic task	579.3	128.9	90.4	6.1	643.9	143.7

### fMRI results

GLM results revealed significant activation in bilateral superior temporal gyrus (STG), middle temporal gyrus (MTG), supplementary motor area (SMA), insular, putamen, and cerebellum during auditory word processing, for both task conditions (PP > Fix and SP > Fix). There was no significant activation when PP was compared to SP (PP > SP). However, SP evoked significant activation in the left inferior frontal gyrus (p. Triangularis and p. Orbitalis), superior medial gyrus, superior frontal gyrus, SMA, and right cerebellum compared to PP (SP > PP). The results are summarized in S3 Fig.

**Fig 2:**
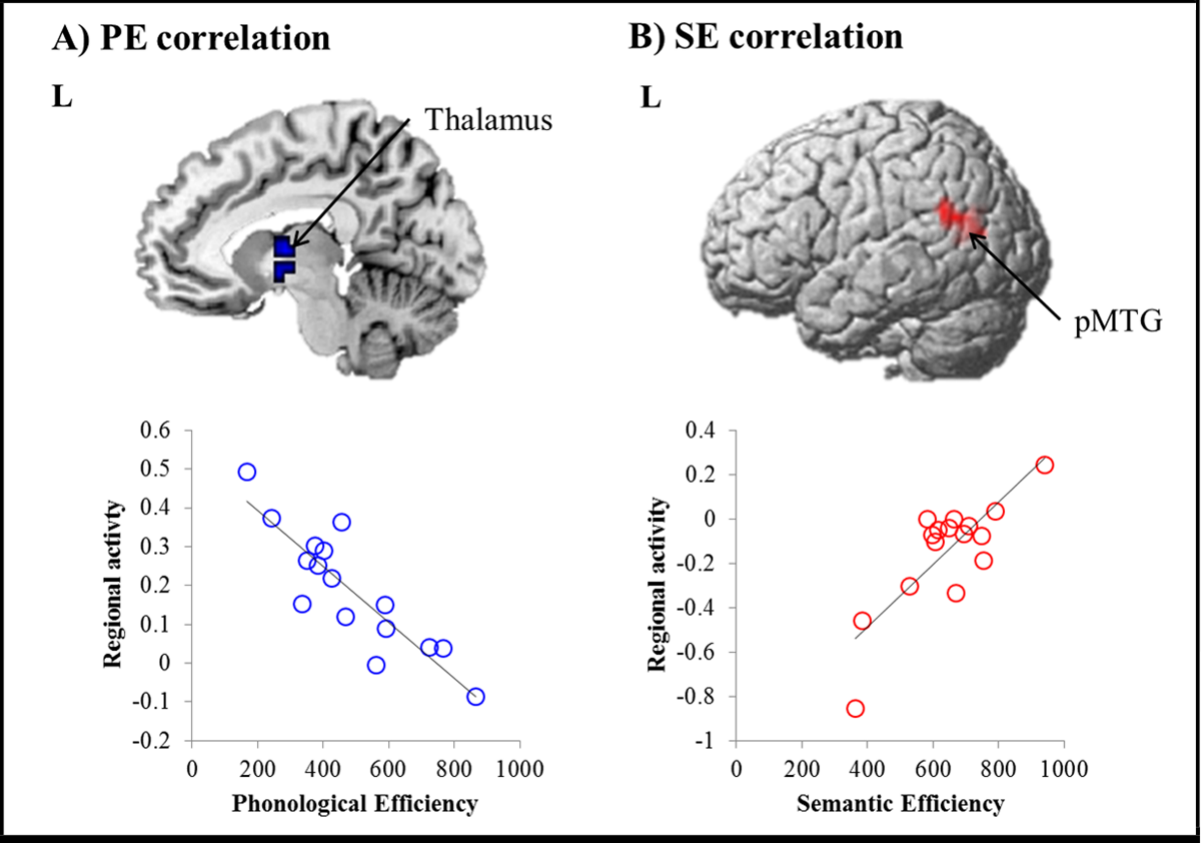
Statistical map of fMRI correlating with inverse efficiency. A) Phonological efficiency (PE) is correlated with the left thalamus negatively. B) Semantic efficiency (SE) is correlated with the left pMTG positively. Circles indicate individuals.

To explore the association between each word processing efficiency and task-induced regional brain activity, we correlated each efficiency values with the each voxel across the whole brain in accordance with task conditions. The left thalamus was negatively correlated with participant’s phonological performance (p _FDR-corrected_ < 0.05). Greater activation of thalamus predicted better phonological performance. Semantic efficiency showed a strong positive correlation with the left pMTG (p _FDR-corrected_ < 0.001). In other words, better performance for semantic processing was associated with lower activation of the left pMTG. These results are summarised in Table 2 and Fig 2.

**Table 2:** Correlation with PE and SE in fMRI data.

				Peak voxel coordinate			
Correlation	Regions	Cluster	Side	x	y	z	Z-score
PE	Thalamus	138	L	-9	-4	6	3.89
			L	-9	-1	-6	3.27
SE	pMTG	290	L	-57	-55	22	4.16
			L	-48	-61	22	3.82

Our analysis demonstrated that two brain regions are associated with the phonological and semantic word processing efficiency separately: thalamus and pMTG. Although we showed the same results using a different baseline, not the fixation (S2 Fig), it would be more appropriate and stringent to use each task as a baseline for each task (phonological: PP > SP; semantic: SP > PP). We performed the same correlational analysis with these contrasts and found no significant voxels survived with the threshold (p FDR-corrected < 0.05, ks > 30). In order to confirm our finding, we performed the ROI analysis on the contrast of PP > SP and SP > PP. ROIs were created as a 8mm sphere based on our original findings (thalamus: MNI [–9 –4 6], pMTG: MNI [–57 –55 22]). We extracted thalamus activity from the contrast of PP > SP and correlated with the phonological efficiency and did same analysis for pMTG activity from the contrast of SP > PP with the semantic efficiency. The results showed that regional activity of thalamus was significantly correlated with PE (r = -0.51, p < 0.05) and pMTG activity was positively associated with SE (r = 0. 53, p < 0.05) (S4 Fig).

### Functional connectivity results

**Fig 3:**
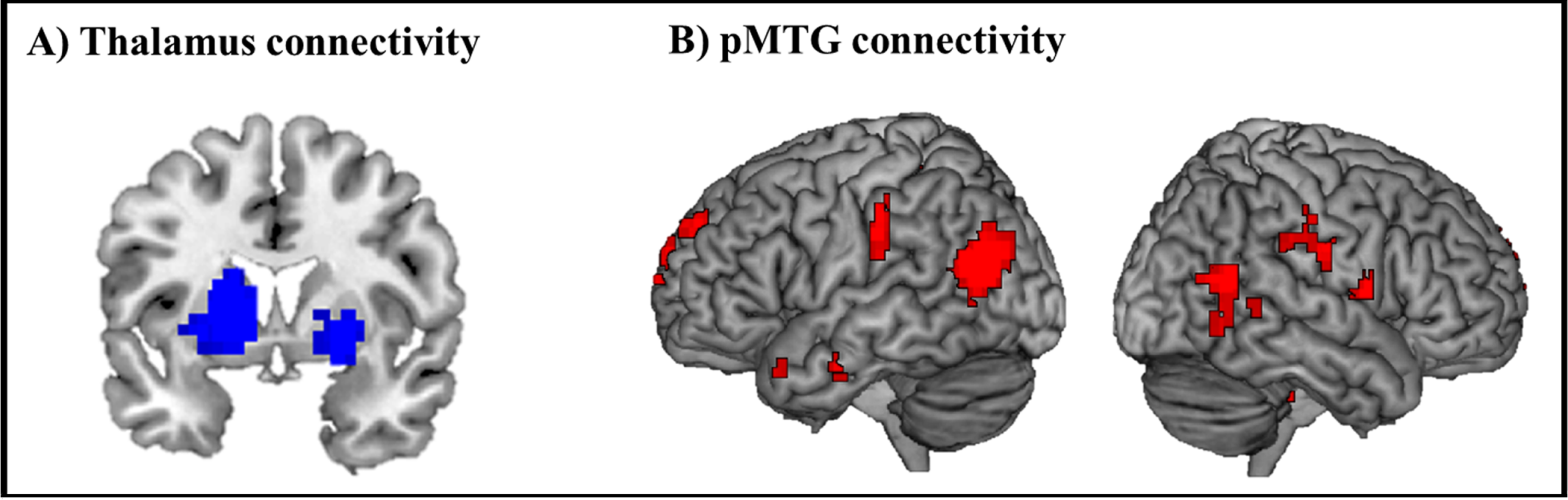
Statistical map of functional connectivity with word efficiency. A) Brain areas are functionally connected to the thalamus as the seed during phonological processing. B) Brain areas are connected to the left pMTG as the seed during semantic processing.

We conducted a seed-based functional connectivity analysis to explore whether the brain areas found in fMRI analyses functioned in cooperation with other brain regions for word processing efficiency. The thalamus and pMTG were used as a seed and correlated with all other voxels in the brain and each efficiency values. The functional connectivity map correlating with PE included the bilateral thalamus (Fig 3A). Many brain regions showed significant functional connectivity with the pMTG. The functional connectivity of SE showed a significant map of pMTG, postcentral gyrus, supramarginal gyrus, and DLPFC (p _FWE_ < 0.05) (Fig 3B). S2 Table summarized the results.

### Structural correlate of word processing efficiency

**Fig 4:**
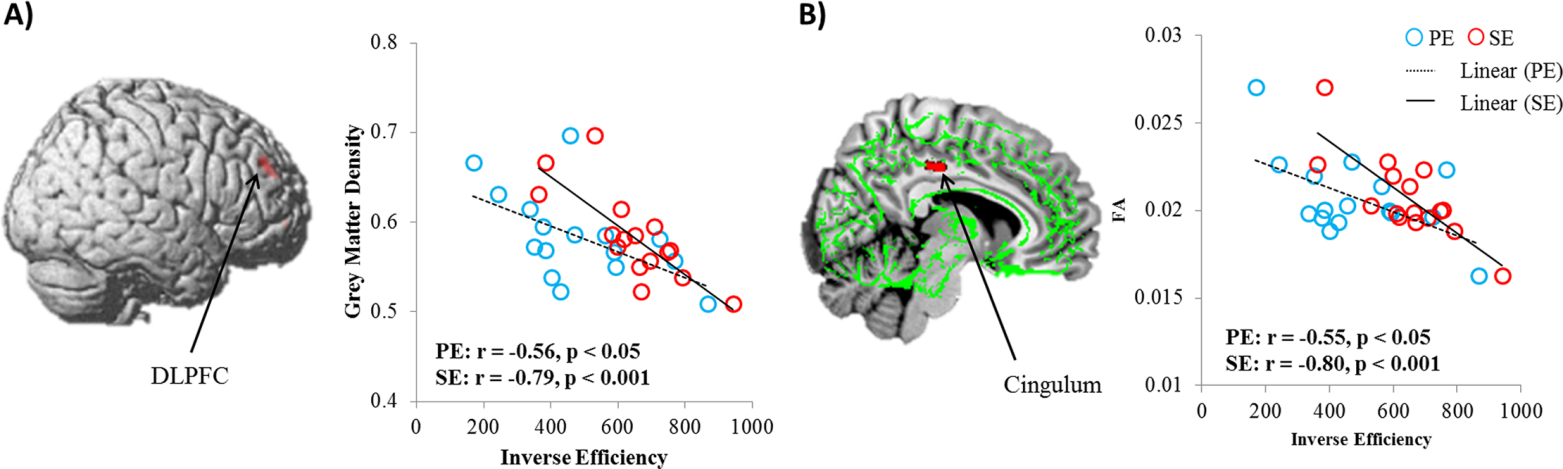
Structural correlation map of semantic efficiency. A) The right DLPFC showed the negative correlation with semantic efficiency. The scatter plot demonstrates that the gray matter density of DLPFC is associated with both inverse efficiency values. B) The right cingulum is negatively correlated with semantic efficiency. The scatter plot shows that the FA of cingulum is significantly correlated with both phonological and semantic efficiency. Blue circles represent participants’ phonological inverse efficiency. Red circles represent participant’s semantic inverse efficiency.

To assess the relationship between word processing efficiency and brain structure, we performed VBM analysis for gray matter and TBSS analysis for white matter. VBM analysis revealed that the right DLPFC was negatively correlated with SE (p _uncorrected_ < 0.001). TBSS showed a negative correlation between the right cingulum and SE (p _uncorrected_ < 0.005). There was no region correlated with PE significantly. To confirm our finding, we correlated each efficiency value with the gray matter density of DLPFC and the FA value of cingulum. Participants with better performance had greater gray matter density in DLPFC than those who with poorer performance (PE r = -0.56, p < 0.05; SE r = -0.79, p <0.001). Higher FA in cingulum was significantly correlated with better performance (PE r = -0.55, p < 0.05; SE r = -0.80, p < 0.001). These results are illustrated in Table 3 and Fig 4.

**Table 3:** Correlation with SE in VBM and DTI data.

				Peak voxel coordinate			
	Regions	Cluster	Side	x	y	z	P values
VBM	DLPFC	158	R	29	45	33	< 0.001
				27	51	25	
DTI	Cingulum	10	R	6	-23	40	< 0.005

### Word processing efficiency in other language regions

**Fig 5:**
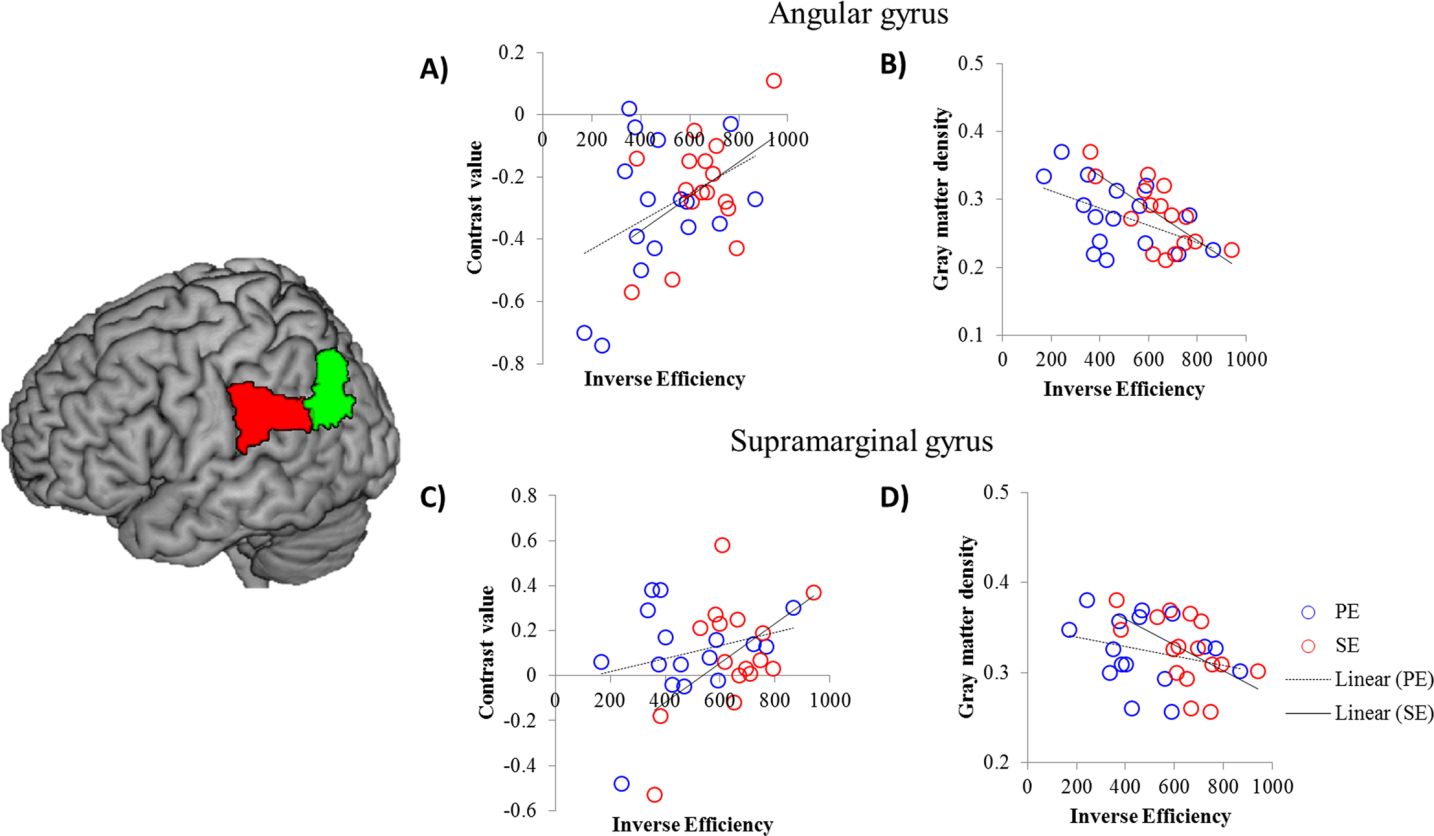
The results of ROI analysis of the supramarginal gyrus and angular gyrus. A) The activity of the angular gyrus showed a trend of a positive correlation with the both efficiencies. B) The GM volume of angular gyrus was negatively correlated with the PE and SE. C) The regional activation of the supramarginal gyrus was correlated with the SE. D) The GM volume of the supramarginal gyrus was negatively correlated with the SE. The red coloured brain region indicates the supramarginal gyrus and the green one, the angular gyrus. The blue circle represents the individual PE and the red ones, the SE.

We conducted ROI analysis by correlating each efficiency value with brain activity, gray matter density, and FA in ROIs (S3 Table). Fig 5 summarized the results of ROI analysis. From fMRI data, only supramarignal gyrus was significantly correlated with SE (r = 0.50, p < 0.05). The gray matter density of supramarginal gyrus also showed a negative correlation with SE significantly (r = -0.53, p < 0.05). The volume of angular gyus was strongly associated with both PE (r = -0.49, p = 0.052) and SE (r = -0.71, p < 0.05). DLPFC showed a marginally significant correlation with PE from VBM (r = -0.49, p = 0.052). There was no difference between PE and SE correlation through all ROIs, which suggested the trend of correlation for both efficiency were not different. There was no significant relationship between the frontal regions and word processing efficiency. No significant correlation was found in FA.

## Discussion

### Functional correlates of word processing efficiency

The present study shows the neural correlates of auditory word processing efficiency in phonological and semantic aspects. Functional imaging results showed that there were two brain areas associated with the phonological and semantic word processing efficiency separately: thalamus and pMTG.

Participants with better performance in the phonological task showed greater activity in the left thalamus. Thalamus has been considered as a gateway of sensory input by transferring information from the sensory periphery to primary neocortical areas [36, 37]. Especially, medial geniculate body, so called the auditory thalamus, is specialized in auditory processing by projecting auditory information to a primary auditory cortex [38]. However, our result demonstrated that the anterior medial part of the left thalamus was associated with the phonological word processing efficiency. Evidence from electrical stimulation studies has reported that the left thalamus plays a special role in language and verbal memory [39–42]. By stimulating thalamus during object naming, they found that the left anterior thalamus was involved in specific alerting response, which is characterized by the acceleration of language and memory processes [39, 41]. The left thalamus functions like a gate to access verbal memory during the tasks and results in changes in language functions for comprehension [43] and production. To perform phonological task efficiently, it can be beneficial to access verbal short-term memory. Thus, our finding can be explained by the role of language and memory function in the left anterior thalamus rather than the auditory processing pathway of the thalamus.

Semantic efficiency was associated with the left pMTG activation. The pMTG has been found a key region in the language comprehension network across modalities such as written words and pictures [44–46], and auditory words [2]. Patient studies with a stroke or semantic dementia also indicated the critical role of the left pMTG for the word level comprehension [47, 48]. Turken and Dronkers [49] recently demonstrated that the left pMTG has extensive structural and functional connectivity with other brain areas related to comprehension and suggested that it plays a crucial role in language comprehension. The left pMTG activation has been observed in the comparison of semantic information relative to baseline [50]. Different from previous studies contrasting semantic condition to baseline, we correlated participants’ semantic performance with their brain activity. Our data revealed that less activity of the left pMTG predicted better performance in the semantic judgment task. A recent meta-analysis [51] demonstrated that the left pMTG was involved in high semantic control. By comparing tasks demanding high semantic control with those demanding low semantic control, they showed that the left pMTG was specialized for high semantic control. Our result can be compatible with their findings in support of the neural efficiency hypothesis [9]. If higher semantic control demands more involvement of the left pMTG, it can be applicable for the case that individuals with low semantic efficiency also require greater activity in the pMTG during the semantic processing. Recently, Wei and colleagues [7] showed the left pMTG is a key region in semantic processing efficiency using resting-state fMRI. They demonstrated that higher regional activity amplitude of the left pMTG at rest predicted better semantic performance. Our result showed the opposite pattern that less activity of the left pMTG was associated with better semantic efficiency during the semantic judgment task. These contrary findings might suggest a possibility of the state-dependent activity of the left pMTG. To explore this possibility, further studies will be needed.

Our results showed that that better phonological performance was associated with higher activities in the thalamus, whereas better semantic performance with less activation in the pMTG. It is still not clear how the neural responses are associated with task performance. But it seems to depend on various factors such as brain areas, task types, cognitive domains and etc. Previous studies examined sensory/motor cortex and related task performance demonstrated that bigger regional activity predicted better sensory function [52, 53]. Especially, Ress et al [52] demonstrated that stronger activity in the primary visual cortex predicted better performance in a visual detection task. The authors suggest that as the fMRI signal is roughly proportional to average firing rates [54, 55], task-induced neural activity in the visual cortex can be the best predictor for their task performance. Similar to other sensory domains, we found that bigger thalamus activity predicted better phonological task performance. Moreover, when we applied less stringent threshold (p unc < 0.01) for the phonological efficiency, there were significant activation in bilateral thalamus, basal ganglia, and the right Heschl’s gyrus–key regions of auditory processing and speech perception [56, 57]. It should be noted that our results (p FDR-corrected < 0.05) showed that the left anterior thalamus was the key region for the phonological word efficiency. As mentioned above, this area plays a role functions like a gate to access verbal working memory during the tasks and results in changes in language functions for comprehension [43]. Working memory function is associated with executive network [58]: more demanding task requires bigger activation of brain areas in the executive network [59] and stronger activation in these regions predicted better task performance [60]. Thus, stronger neural activity in thalamus may be beneficial for phonological efficiency. Taken altogether, the brain areas, task types, and cognitive domains can contribute to the discrepancy in neural responses associated with thalamus and pMTG.

### Structural correlates of word processing efficiency

VBM results exhibited that greater volume of the right DLPFC predicted better word processing performance. Generally, DLPFC is described as an executive centre for directing attention, working memory, and decision making [61, 62]. Especially, the right DLPFC activation was found in monitoring and controlling in task execution [63]. Another study showed that the performance of language ability was positively correlated with increased the right DLPFC activation [64]. Our finding is consistent with these functional evidences by revealing that individuals with better performance in word processing showed greater volume in the right DLPFC. It suggests a role of DLPFC, monitoring in the present task to aid language function.

TBSS results showed that the cingulum was associated with the performance of word processing. Anatomically, cingulum is a bundle of white matter projecting from cingulate gyrus to entorhinal cortex [65]. The posterior part of it is related to cognitive functions such as attention and memory. Patients with Alzheimer or mild cognitive impairment showed significantly reduced FA in the posterior cingulum compared to healthy controls [66, 67]. Kantarci and colleagues [68] reported that the posterior cingulum FA was associated with cognitive functions including language, attention, and executive functions in elderly population. In accordance with previous findings, our data also demonstrated that higher posterior cingulum FA predicted better performance in language function.

Our data showed that structural correlates of word processing efficiency were attributed to brain regions related to general cognitive function, not language specific regions. Individual differences in a specific function can be embedded in their general cognitive ability such as intelligence [69]. Thus, it suggests brain areas involved in general cognitive ability may contribute to the task performance of a specific domain of cognition such as language.

### Neural efficiency of semantic network

The pMTG network identified here largely overlaps with brain areas have been suggested for semantic processing from functional imaging studies. The functional connectivity maps for the phonological and semantic efficiency overlapped with each other by revealing that the left pMTG was functionally connected with the left DLPFC, left ATL, and right pMTG for word processing efficiency. All regions in our functional connectivity results were reported in previous meta-analysis of semantic processing from fMRI and positron emission tomography [51, 70]. A study correlating individuals’ semantic performance with their resting-state fMRI also showed similar functional connectivity maps [7]. They used the left pMTG as seed region and correlated the seed with other regions in a whole brain as well as participants’ semantic performance score. They found the functional connectivity of semantic network including bilateral pMTG, ATL, posterior cingulate gyrus, DLPFC, and IFG predicted individuals’ performance. Even if the network observed here highly overlaps with the semantic network, it is noted that the characteristic of our network showed neural efficiency for word processing. All areas in the network were positively correlated with word efficiency, suggesting participants with poorer performance activated this network greater, in support of the neural efficiency hypothesis [71]. In semantic network, there is no previous study to support this neural efficiency hypothesis yet. However, a recent meta-analysis study provided a promising possibility by demonstrating tasks demanding high semantic control activated semantic network significantly more than tasks with low semantic control [51].

### Word processing efficiency in other language regions

We explored potential relationships between word processing efficiency and language related brain regions. Supramarginal gyrus and angular gyus showed functional and structural correlations with word processing efficiency. Supramarginal gyrus is known as one of perisylvian language regions and is involved in phonological processing in language function [72–74]. However, our correlation results showed that supramarginal gyrus was associated with semantic efficiency more than phonological efficiency. Structural imaging studies have suggested that supramarginal gyus is associated with word knowledge in language processing [13–15]. Lee and colleagues [13] showed that gray matter density of this region was positively correlated with word knowledge. Structural and functional association of suprmarginal gyus with semantic processing efficiency at current study replicated these findings and might suggest its supportive role of general word knowledge.

Angular gyrus has been observed in relation to various cognitive functions including language, attention, memory retrieval, spatial cognition, and number processing [75], by suggesting its role of complex information integration and knowledge retrieval [70]. Functional imaging studies demonstrated that activation of this region has been found in semantic processing consistently [45, 70, 76, 77]. Our finding also replicated previous finding by showing functional and structural correlation between angular gyrus and semantic word processing efficiency. Overall, language areas in parietal lobe participated in word processing and contributed to its efficiency in language comprehension structurally and functionally.

### Limitations

Although we performed the power analysis to determine the number of subject, it should be acknowledged that the sample size is relatively small in this study. Button and colleagues [78] pointed out that most of neuroscience studies had very low statistical power due to the small sample size and it would result in higher risk to obtain false negative as well as false positive results. Therefore, our results should be interpreted with a caution and future work is needed to replicate and generalize our findings.

## Conclusions

In conclusion, we observed two brain regions playing significant roles in phonological and semantic efficiency. The functional activity of the left thalamus was associated with phonological performance, whereas the level of activity in the left pMTG predicted semantic performance; Individuals with stronger activity in thalamus and less activity in pMTG showed better word processing efficiency. These findings implicate that two aspects of word processing, phonology or semantics, are served in different brain areas with different manners. The functional network related to pMTG was overlapped with a distributed large-scale of semantic network, which contributed to word processing efficiency in a neural efficient manner. Also, language regions in parietal lobe also supported the idea that the subsets of language network play a role in word processing efficiency. Furthermore, DLPFC and cingulum were associated with both phonological and semantic efficiency. We conclude that the subsets of language networks as well as general cognitive regions contribute to the word processing efficiency functionally and structurally.

## Supporting information

S1 Fig Individual task performance. (DOCX)

S2 Fig Statistical map of fMRI correlating with PE and SE in comparison with a control task from Visser et al (2011). (DOCX)

S3 Fig GLM results. (DOCX)

S4 Fig The results of ROI analysis. (DOCX)

S1 Table The list of stimuli. (DOCX)

S2 Table Functional connectivity map of word efficiency. (DOCX)

S3 Table The results of ROI correlation analysis. (DOCX)
