# Supplementary material for: Structural and functional correlates for language efficiency in auditory word processing
Source: PLoS One. 2017 Sep 11;12(9):e0184232. doi: 10.1371/journal.pone.0184232 (PMC5593184; doi:10.1371/journal.pone.0184232)
Supplement: S1 Fig — (DOCX) [file pone.0184232.s001.docx]

**S1 Fig.**


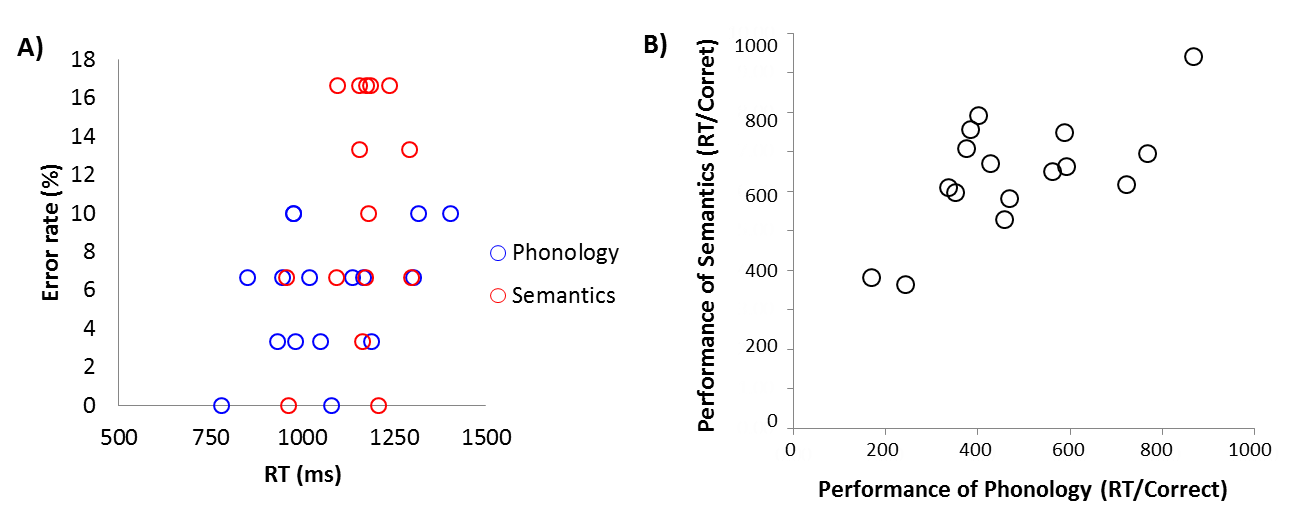


S1 Fig. Individual task performance. A) A scatter plot shows individuals’ performance for the phonology (blue circle) and semantic (red circle). B) The relationship between the phonological task performance and semantic task performance. The task performance (efficiency) was calculated by dividing the average response time of correct trials by accuracy.
