# Supplementary material for: Structural and functional correlates for language efficiency in auditory word processing
Source: PLoS One. 2017 Sep 11;12(9):e0184232. doi: 10.1371/journal.pone.0184232 (PMC5593184; doi:10.1371/journal.pone.0184232)
Supplement: S2 Fig — (DOCX) [file pone.0184232.s002.docx]

**S2 Fig.**


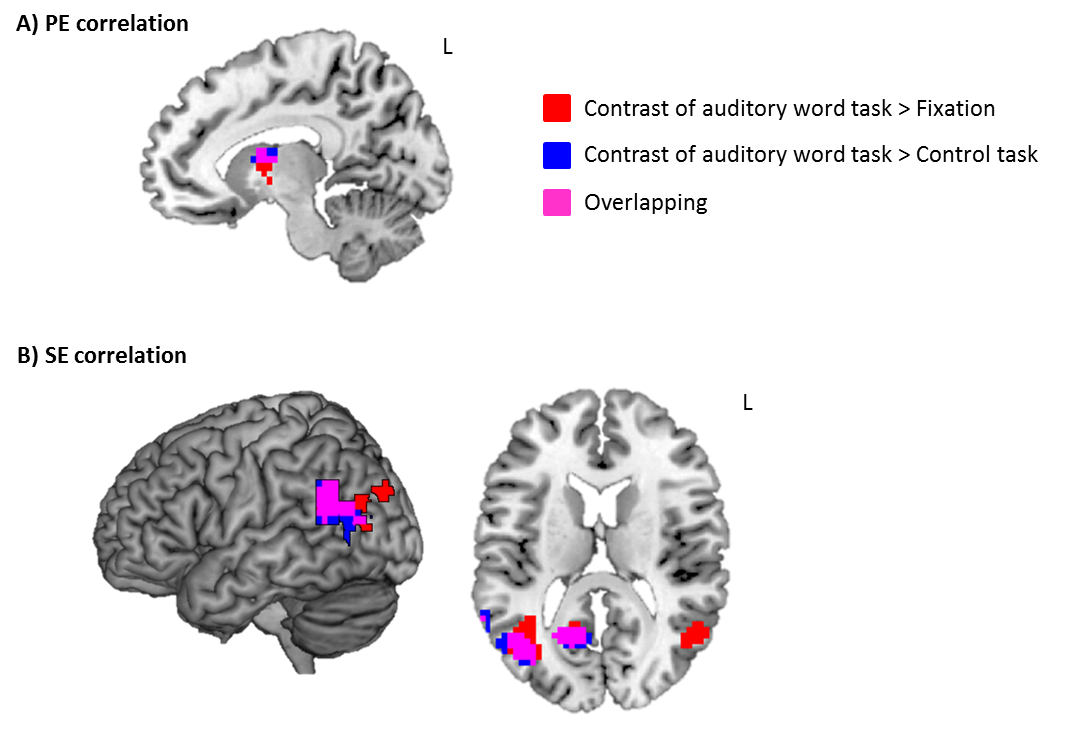


S2 Fig. Statistical map of fMRI correlating with PE and SE in comparison with a control task from Visser et al (2011). A) Phonological efficiency (PE) is correlated with left thalamus negatively. B) Semantic efficiency (SE) is correlated with left pMTG and cuenus positively. Red coloured regions showed the results from the comparisons between word tasks and fixation. Blue coloured regions showed the results from the comparisons between word tasks and the control task. Pink colour indicates the overlapping regions.
