# Supplementary material for: Structural and functional correlates for language efficiency in auditory word processing
Source: PLoS One. 2017 Sep 11;12(9):e0184232. doi: 10.1371/journal.pone.0184232 (PMC5593184; doi:10.1371/journal.pone.0184232)
Supplement: S3 Fig — (DOCX) [file pone.0184232.s003.docx]

**S3 Fig**

**
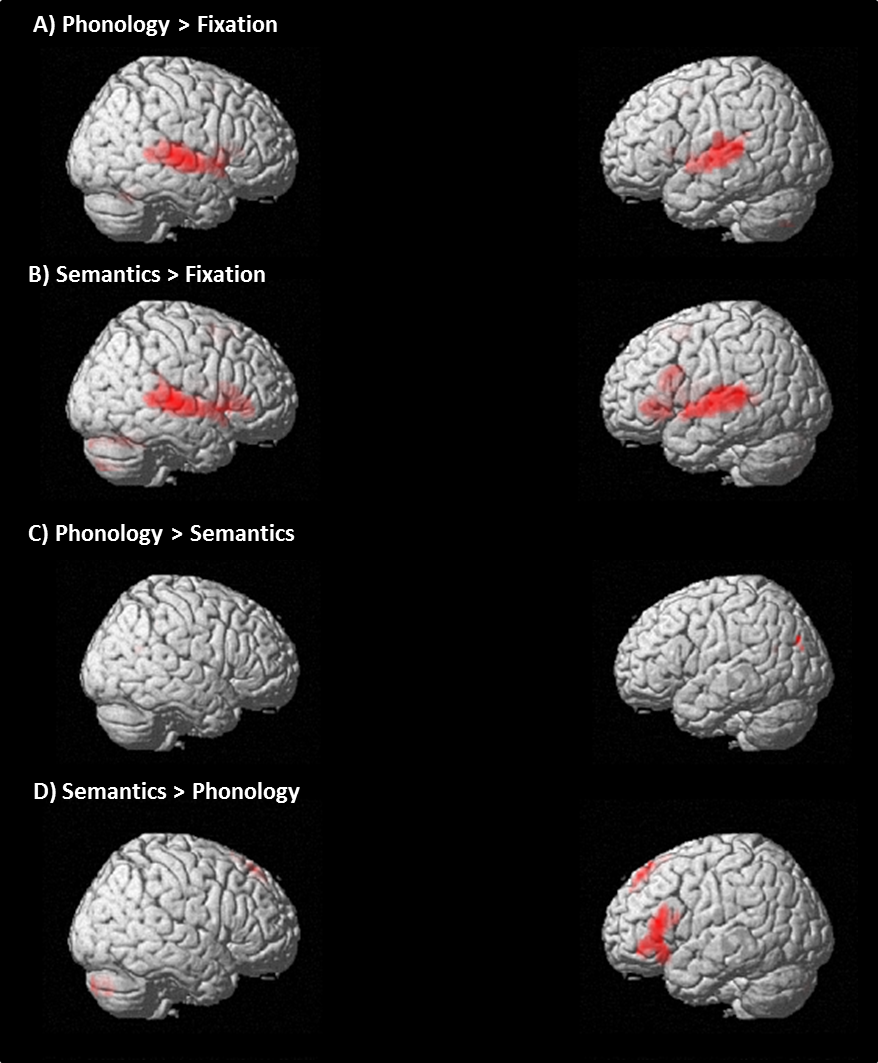
**

**S3 Fig. GLM results.** A) Brain activation map of the contrast PP > Fix. B) Brain activation map of the contrast SP > Fix. C) Brain activation map of the contrast PP > SP. D) Brain activation map of the contrast SP > PP.
