# Supplementary figures and images for: Structural and functional correlates for language efficiency in auditory word processing
Source: PLoS One. 2017 Sep 11;12(9):e0184232. doi: 10.1371/journal.pone.0184232 (PMC5593184; doi:10.1371/journal.pone.0184232)

**S4 Fig.**

**
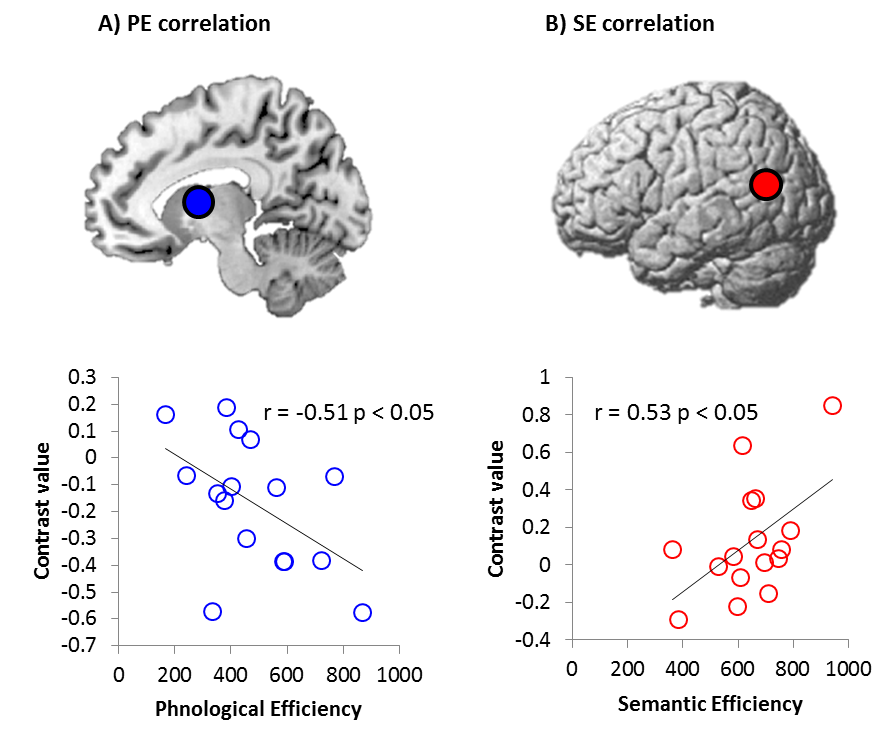
**

**S4 Fig. The results of ROI analysis.**

Supplement: S4 Fig — (DOCX) [file pone.0184232.s004.docx]
