# Supplementary material for: Structural and functional correlates for language efficiency in auditory word processing
Source: PLoS One. 2017 Sep 11;12(9):e0184232. doi: 10.1371/journal.pone.0184232 (PMC5593184; doi:10.1371/journal.pone.0184232)
Supplement: S1 Table — (DOCX) [file pone.0184232.s005.docx]

**S1 Table**

| Condition | Item | Meaning | Condition | Item | Meaning |
| --- | --- | --- | --- | --- | --- |
| Phonological processing | 가격 | price | Semantic Processing | 가:짜 | fake |
|  | 무릎 | knee |  | 거:절 | refusal |
|  | 비누 | soup |  | 도박 | gambling |
|  | 소금 | salt |  | 대:립 | conflict |
|  | 사진 | picture |  | 뇌물 | bribe |
|  | 가방 | bag |  | 도둑 | thief |
|  | 시간 | time |  | 비:극 | tragedy |
|  | 자리 | room |  | 사:망 | death |
|  | 머리 | head |  | 시:련 | ordeal |
|  | 소리 | sound |  | 무덤 | grave |
|  | 부엌 | kitchen |  | 자살 | suicide |
|  | 가족 | family |  | 거:지 | beggar |
|  | 기름 | oil |  | 노:화 | aging |
|  | 바지 | pants |  | 고통 | pain |
|  | 저녁 | evening |  | 비:만 | obesity |
|  | 비:서 | secretary |  | 미:모 | beauty |
|  | 사:십 | forty |  | 다행 | lucky |
|  | 자:세 | posture |  | 사랑 | love |
|  | 자:랑 | boast |  | 화해 | reconciliation |
|  | 수:건 | towel |  | 가:망 | prospect |
|  | 비:밀 | secret |  | 사탕 | candy |
|  | 가:게 | store |  | 다정 | tenderness |
|  | 서:술 | description |  | 저:택 | mansion |
|  | 세:상 | world |  | 미:덕 | virtue |
|  | 주:소 | address |  | 저:축 | saving |
|  | 서:론 | introduction |  | 고향 | hometown |
|  | 수:박 | watermelon |  | 마:법 | magic |
|  | 도:착 | arrival |  | 새벽 | dawn |
|  | 소:변 | urine |  | 희망 | hope |
|  | 비:교 | comparison |  | 노력 | effort |

**S1 Table The list of stimuli**
