# Supplementary material for: Structural and functional correlates for language efficiency in auditory word processing
Source: PLoS One. 2017 Sep 11;12(9):e0184232. doi: 10.1371/journal.pone.0184232 (PMC5593184; doi:10.1371/journal.pone.0184232)
Supplement: S2 Table — (DOCX) [file pone.0184232.s006.docx]

**S2 Table**

|  |  |  |  | Peak voxel coordinate | | |  |
| --- | --- | --- | --- | --- | --- | --- | --- |
|  | Regions | Cluster | Side | x | y | z | Z-score |
| PE | Thalamus | 1337 | L | -12 | -2 | 10 | 5.77 |
|  |  |  |  | -40 | -60 | 24 | 5.48 |
|  |  | 360 | R | 20 | 4 | -8 | 4.66 |
| SE | pMTG | 1564 | L | -44 | -64 | 14 | 7.11 |
|  |  | 246 | R | 58 | -56 | 14 | 4.01 |
|  |  |  |  | 50 | -52 | 14 | 3.9 |
|  | Supramariginal gyrus | 189 | R | 58 | -26 | 32 | 4.06 |
|  |  |  |  | 50 | -28 | 28 | 3.9 |
|  | Postcentral gyrus |  |  | 68 | -14 | 28 | 4.06 |
|  | DLPFC | 128 | L | -8 | 64 | 24 | 3.98 |
|  |  |  |  | -20 | 52 | 36 | 3.51 |

**S2 Table** Functional connectivity map of word efficiency
