# Supplementary material for: Structural and functional correlates for language efficiency in auditory word processing
Source: PLoS One. 2017 Sep 11;12(9):e0184232. doi: 10.1371/journal.pone.0184232 (PMC5593184; doi:10.1371/journal.pone.0184232)
Supplement: S3 Table — (DOCX) [file pone.0184232.s007.docx]

**S3 Table**

|  |  | PE | |  | SE | |
| --- | --- | --- | --- | --- | --- | --- |
|  | Region | r | p |  | r | p |
| fMRI | Frontal lobe |  |  |  |  |  |
|  | DLPFC | -0.053 | 0.845 |  | 0.306 | 0.249 |
|  | p. Orbitalis | 0.072 | 0.791 |  | 0.266 | 0.32 |
|  | p. Triangularis | 0.003 | 0.99 |  | 0.179 | 0.507 |
|  | p. Opercularis | -0.01 | 0.969 |  | 0.227 | 0.398 |
|  | Parietal lobe |  |  |  |  |  |
|  | Supramarginal G | 0.265 | 0.32 |  | **0.502** | **0.047** |
|  | Angular G | 0.383 | 0.143 |  | 0.46 | 0.073 |
| VBM | Frontal lobe |  |  |  |  |  |
|  | DLPFC | -0.493 | 0.052 |  | -0.329 | 0.214 |
|  | p. Orbitalis | -0.37 | 0.158 |  | -0.307 | 0.247 |
|  | p. Triangularis | -0.216 | 0.422 |  | -0.21 | 0.435 |
|  | p. Opercularis | -0.343 | 0.194 |  | -0.086 | 0.751 |
|  | Parietal lobe |  |  |  |  |  |
|  | Supramarginal G | -0.294 | 0.269 |  | **-0.553** | **0.026** |
|  | Angular G | -0.494 | 0.052 |  | **-0.705** | **0.002** |
| DTI | White matter tracts |  |  |  |  |  |
|  | Uncinate F | -0.392 | 0.133 |  | 0.164 | 0.544 |
|  | External capsule | -0.231 | 0.39 |  | 0.294 | 0.269 |
|  | Superior longitudinal F | 0.15 | 0.579 |  | 0.466 | 0.069 |
|  | Sagittal stratum | -0.23 | 0.391 |  | 0.086 | 0.752 |

**S3** **Table** The results of ROI correlation analysis. r represents Pearson’s coefficient. The numbers marked in bold type indicate significant correlations.
